# Prognostic Role of Basal Serum Alpha-Fetoprotein in Patients with Hepatocellular Carcinoma Suitable for Curative Treatment

**DOI:** 10.3390/medicina60050692

**Published:** 2024-04-24

**Authors:** Stefano Mazza, Chiara Frigerio, Daniele Alfieri, Aurelio Mauro, Francesca Torello Viera, Davide Scalvini, Chiara Barteselli, Carmelo Sgarlata, Letizia Veronese, Marco Bardone, Laura Rovedatti, Simona Agazzi, Elena Strada, Lodovica Pozzi, Marcello Maestri, Valentina Ravetta, Andrea Anderloni

**Affiliations:** 1 Gastroenterology and Endoscopy Unit, Fondazione IRCCS Policlinico San Matteo, 27100 Pavia, Italy; 2 Department of Internal Medicine and Therapeutics, University of Pavia, 27100 Pavia, Italy; 3 General Surgery I, Fondazione IRCCS Policlinico San Matteo, 27100 Pavia, Italy

**Keywords:** hepatocellular carcinoma, alpha-fetoprotein, AFP cut-off, survival, prognosis

## Abstract

*Background and Objectives*: Serum alpha-fetoprotein (AFP) is a recognized affordable oncological marker in patients with hepatocellular carcinoma (HCC). However, AFP’s prognostic role has been assessed mainly after specific treatments, and no unanimously recognized cut-offs have been identified. The aim of this study is to investigate the prognostic role of different basal AFP cut-offs on survival and HCC course. *Materials and Methods*: In this single-center, retrospective study, all patients newly diagnosed with HCC between January 2009 and December 2021 were prospectively enrolled. Only patients suitable for curative HCC treatments were included in the analyses. Patients were stratified according to AFP cut-offs of 20, 200, 400, and 1000 ng/mL, which were correlated with survival outcomes and clinical parameters. *Results*: A total of 266 patients were analyzed, with a median follow-up time of 41.5 months. Median overall survival (OS) of all cohort was 43 months. At the multivariate Cox-regression analysis, AFP value ≥ 1000 ng/mL correlated with impaired OS (1-year OS: 67% vs. 88%, 5-year OS: 1% vs. 43%; *p* = 0.005); other risk factors were tumor dimension ≥ 5 cm (HR 1.73; *p* = 0.002), Child–Pugh class B–C (HR 1.72; *p* = 0.002), BCLC stage A (vs. 0) (HR 2.4; *p* = 0.011), and malignant portal vein thrombosis (HR 2.57; *p* = 0.007). AFP ≥ 1000 ng/mL was also associated with a reduced recurrence-free survival (HR 2.0; *p* = 0.038), while starting from AFP ≥ 20 ng/mL, a correlation with development of HCC metastases over time (HR 3.5; *p* = 0.002) was seen. AFP values ≥ 20 ng/mL significantly correlated with tumor size and higher histological grading; starting from AFP values ≥ 400 ng/mL, a significant correlation with Child–Pugh class B–C and female gender was also observed. *Conclusions*: Basal AFP correlates with relevant outcomes in patients with HCC. It could help identify patients at a higher risk of worse prognosis who might benefit from personalized surveillance and treatment programs. Prospective studies are needed to confirm these results.

## 1. Introduction

Hepatocellular carcinoma (HCC) accounts for more than 80% of primary liver tumors [1,2], representing the seventh most common cancer and the fourth cause of cancer-related death worldwide [3]. The main risk factors are hepatitis B virus, hepatitis C virus, alcohol abuse determining alcoholic steatohepatitis, and nonalcoholic fatty liver disease/steatohepatitis [4,5]. Most HCC cases, approximately 80%, develop from liver cirrhosis, HCC being the most common cirrhosis complication [6]. Such a high incidence of HCC in cirrhotic patients supports the implementation of surveillance programs, with a 6-month surveillance with abdominal ultrasound being recommended by European and American guidelines [7,8]. The gold standard for HCC diagnosis and follow-up in cirrhotic patients is imaging methods, especially contrast-enhanced ultrasound (CEUS), computed tomography (CT), and contrast-enhanced magnetic resonance (MRI) [9]. HCC that arises in the context of cirrhosis is staged not solely based on the extent of the disease but also by considering the degree of liver function, which represents an indispensable parameter for prognostic purposes and choice of treatment [10]. The Barcelona Clinic for Liver Cancer (BCLC) algorithm is the most widely used HCC staging system, which also provides a first-line therapeutic indication for each stage [11]. The prognosis of HCC depends significantly on the staging of the disease and the suitable treatment options; however, these staging systems are suboptimal, and several studies have underlined how other biological markers are associated with tumor prognosis. Additionally, despite improvements in therapeutic approaches, long-term survival remains poor, with 5-year and 10-year survival rates of about 20% and 10%, respectively [10].

Among HCC-related biomarkers, alpha-fetoprotein (AFP) is an oncofetal glycoprotein which is normally produced by the yolk sac in the first trimester of pregnancy and by the fetal liver starting from the eleventh to twelfth week of gestation [12]. In adult life, the production of AFP is drastically reduced and can be measured in minimal concentrations in healthy adults, between 3 and 20 ng/mL [13,14]. The use of AFP as an oncological marker of HCC was first proposed in the 1960s, though AFP is not specific for HCC, as numerous neoplastic and non-neoplastic conditions involving the liver and other organs can cause an increase in it [15]. Despite the cut-off value of 20 ng/mL being commonly used, some studies have shown an increase in specificity for HCC diagnosis by raising the cut-off to 200 ng/mL, and especially 400 ng/mL [16]; however, most small HCC at diagnoses arise in patients with negative serum AFP [17,18,19].

Besides the limited value of AFP for diagnosis, the value of AFP as a prognostic is gaining ground. Indeed, AFP monitoring is very useful for evaluating the response to a specific treatment [20], and patients with low or negative AFP have a lower probability of post-treatment relapse and increased survival [21,22]. Some recent prognostic scores, such as the CLIP score and the ITA.LI.CA score, also include serum AFP, in addition to other clinical and tumor features [23,24]. However, the prognostic role of AFP in these studies depends on patient characteristics, study design, and the values used as cut-offs [25,26], while several studies, on the other hand, seem not to confirm this role [27]. Moreover, studies assessing the relationship between baseline AFP values and prognostic parameters independently from a specific therapy are few and mainly analyze populations different from the Italian one in terms of ethnicity and the distribution of risk factors [28,29]. 

Based on these premises, our study aimed to assess the role of different baseline AFP cut-offs towards relevant survival outcomes in a cohort of Italian patients with a new diagnosis of HCC suitable for curative treatment.

## 2. Materials and Methods

### 2.1. Study Design and Patients

We performed a single-center, retrospective, observational study. Between January 2009 and December 2021, all patients newly diagnosed with HCC were consecutively enrolled and followed-up over time.

We included only HCC patients deemed suitable for a curative treatment after a multidisciplinary discussion in order to obtain a more homogeneous cohort, avoiding biases related to very different disease prognosis. Therefore, all patients included in this study were classified as BCLC 0 (very early) or BCLC A (early) stage to be suitable for curative treatment. We considered the following as curative approaches: liver resection; radiofrequency thermal ablation (RFTA); and percutaneous ethanol injection (PEI). Some patients also underwent transcatheter arterial chemoembolization (TACE) or transarterial chemoembolization (TAE), but always in combination with RFTA or PEI. No patients received concomitant or adjuvant systemic therapy, given the lack of strong evidence of a benefit from this approach at the enrolment period of our study. No patients in our cohort underwent liver transplantation, because our center is not a referral center for liver transplantation, and therefore, we do not routinely follow-up patients after this kind of treatment.

At enrolment, the following baseline parameters were collected: sex; date of HCC diagnosis; etiology of liver disease; size of the nodule; Child–Pugh score; AFP value; histological grading of HCC; and presence of portal thrombosis (PVT) and if malignant. Regarding the etiology of cirrhosis, the patients were divided into two groups, one with positivity for major hepatotropic viruses (HBV, HCV) and one without positivity for major hepatotropic viruses. For convenience, patients with multifactorial etiology but with evidence of viral infections were included in the first group. A subdivision into two groups was also performed for the size of the tumor lesion, identifying a group with HCC < 5 cm in size and a group with HCC > or equal to 5 cm. 

The patients were then stratified according to the following baseline AFP cut-off values: < or ≥20 ng/mL; < or ≥200 ng/mL; < or ≥400 ng/mL; and < or ≥1000 ng/mL.

For the final inclusion in the study analyses, patients with at least one of the following criteria were excluded: previous diagnosis of HCC; HCC without pre-existing liver cirrhosis; unknown etiology of liver cirrhosis; baseline AFP value not available; mixed HCC-cholangiocarcinoma (CCC) histotype; and unknown data and/or cause of death or loss at follow-up.

This study was approved by the local Ethics Committee and was performed according to the Helsinki Declaration. All patients gave their written informed consent for inclusion in this study and clinical data collection.

### 2.2. HCC Assessment and Follow-Up

HCC diagnosis was made radiologically when typical hallmarks were visible at contrast-enhanced CT and/or MRI, or histologically by means of liver biopsy, according to the European Association for the Study of the Liver and the American Association for the Study of Liver Diseases guidelines in force at the time of diagnosis [7,30]. Tumor dimension was assessed at the cross-sectional imaging technique (CT, MRI, and/or US) performed. The presence of PVT and its possible neoplastic nature were assessed by CEUS.

In patients undergoing liver biopsy, the histological grading of HCC was assigned by a group of onco-pathologists with strong expertise and in accordance with the 2019 World Health Organization (WHO) classification [31].

All patients were followed-up by a multidisciplinary team, including oncologists, gastroenterologists, radiologists, and surgeons, with expertise in the management of liver diseases and HCC, with scheduled outpatient visits and imaging examinations. Specifically, after the treatment a contrast-enhanced CT scan was performed at 1 month, and subsequently every 6 months with CEUS or contrast-enhanced CT scan; if a doubt emerged at CEUS or contrast-enhanced CT, then a contrast-enhanced CT or MRI was performed, respectively. 

### 2.3. Outcome Measures

During the follow-up, the following outcome measures were recorded: death, both considered as overall survival (OS) and disease-specific survival (DSS), and HCC recurrence (recurrence-free survival, RFS), which included local recurrence after treatment, new intrahepatic HCC, and development of extrahepatic HCC metastases over time. For DSS, only death related to the liver disease, namely liver failure or progression of liver cancer, was considered. 

OS, DSS, and RFS were considered as time-dependent variables, and the time was calculated from HCC diagnosis to death or last follow-up for OS and DSS, and from HCC treatment to recurrence or last follow-up for RFS. Local recurrence after treatment, new intrahepatic HCC, and HCC metastasis development were instead considered as time-independent variables for the purpose of analysis. Surviving patients were followed-up for at least 6 months after HCC diagnosis.

### 2.4. Study Objectives

The primary objective of this study was to correlate the different AFP cut-offs with the OS. The secondary objectives were as follows:

- To correlate the different AFP cut-offs with the other outcome measures;
- To correlate the AFP cut-offs with the baseline clinical variables.

### 2.5. Statistical Analysis

The categorical variables were described as absolute frequency and percentage. The continuous variables were described as median and range. Kaplan–Meier curves were computed to assess the survival outcomes, namely the overall survival, the disease-related survival, and the recurrence-free survival. Survival data were expressed as the median survival time and as the survival at 1-year and 5-year time points, with the relevant 95% confidence intervals (CI). The predictive role of baseline clinical variables towards survival outcomes was assessed by means of Cox regression analysis and the results are expressed as hazard ratio (HR) with 95%CI. Specifically, survival analyses were performed calculating the hazard ratio of death or recurrence. Variables significant in the univariate analysis were entered in the multivariate model. Correlations between the different AFP cut-offs and local recurrence, new intrahepatic HCC, and HCC metastasis development were assessed by using the bivariate logistic regression analysis and expressed as hazard ratio (HR) with 95%CI. Correlations between the different AFP cut-offs and other baseline variables were assessed by using the Mann–Whitney analysis and Fisher’s exact tests for continuous and categorical variables, respectively. All the analyses were carried out by computer software IBM SPSS Statistics (Version 25; IBM Corporation, Armonk, NY, USA), and significance was established at the 0.05 level (two-sided).

## 3. Results

### 3.1. Baseline Features

A total of 266 patients newly diagnosed with HCC and eligible for a treatment with curative intent were consecutively enrolled (32% female, median age at enrollment 73 years). The predominant cirrhosis etiology was viral (80%). RFTA was the most frequently performed treatment (76%), followed by PEI (18%); some patients underwent more than one type of treatment. Most patients were in Child–Pugh A class (81%) and had an HCC of grade 1–2 (85%) at diagnosis, while PVT was present in 42 (16%) patients, 37 (14%) of which were of neoplastic nature. Most patients were AFP-negative (<20 ng/mL) at diagnosis (66%): 12% had AFP ≥ 200 ng/mL, 8% ≥ 400 ng/mL, and 6% ≥ 1000 ng/mL. All the demographic and baseline clinical features, both overall and stratified by AFP cut-offs, are reported in Table 1. The median follow-up time was 41.5 months (1–174).

### 3.2. Correlations between AFP and Survival

During the follow-up, 212 patients (79.7%) died, of whom 157 (59.0%) died due to liver-related disease. 

**Figure 1:**
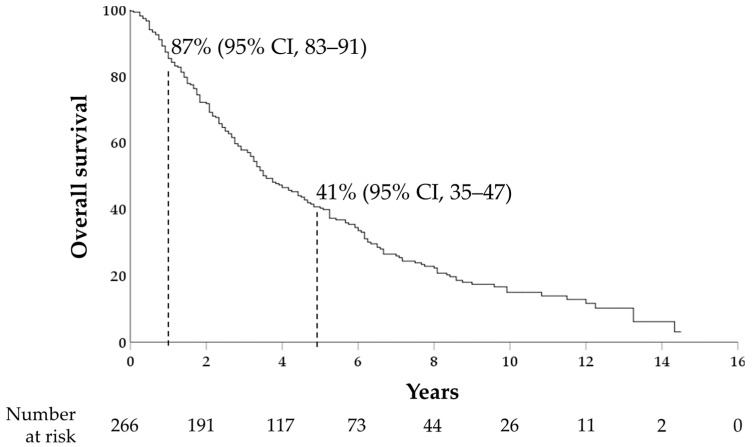
Kaplan–Meier curve for overall survival; 1- and 5-year survival rates are shown.

The 1- and 5-year cumulative probabilities of OS were 87% (95%CI, 83–91) and 41% (95%CI, 35–47), respectively, with a median OS of 43 months (95%CI, 35–51) (Figure 1).

**Figure 2:**
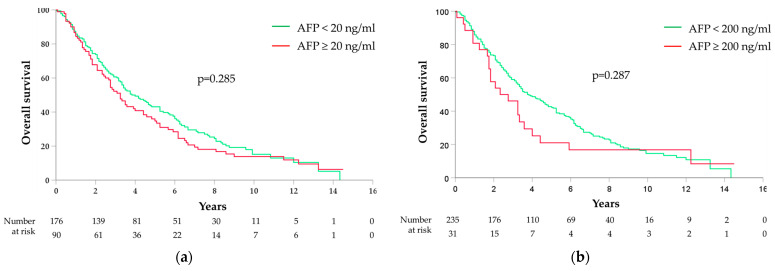

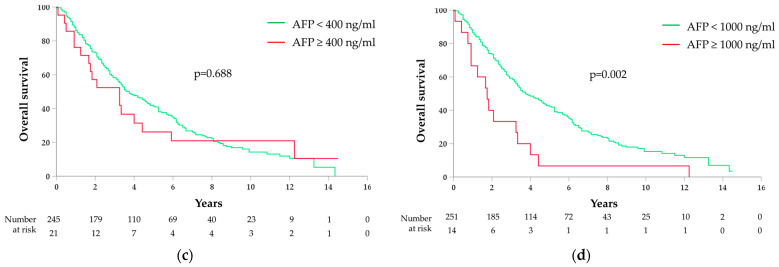
Kaplan–Meier curves for overall survival stratified by AFP cut-offs: (**a**) 20 ng/mL; (**b**) 200 ng/mL; (**c**) 400 ng/mL; (**d**) 1000 ng/mL.

By differentiating between the different AFP cut-offs, no correlation was found between the OS and the AFP cut-offs of 20 ng/mL (median OS 46 months [95%CI 36–56] for AFP < 20 ng/mL and 39 months [95%CI 32–46] for AFP ≥ 20 ng/mL, *p* = 0.285) (Figure 2a); 200 ng/mL (median OS 46 months [95%CI 37–55] for AFP < 200 ng/mL and 28 months [95%CI 12–44] for AFP ≥ 200 ng/mL, *p* = 0.287) (Figure 2b); and 400 ng/mL (median OS 45 months [95%CI 36–54] for AFP < 400 ng/mL and 39 months [95%CI 15–63] for AFP ≥ 400 ng/mL, *p* = 0.688) (Figure 2c). 

A significant correlation was instead observed between the OS and the AFP cut-off of 1000 ng/mL (HR 2.3; 95%CI 1.3–3.9; *p* = 0.002), with a median OS of 45 months (95%CI 36–54) for AFP < 1000 ng/mL and 21 months (95%CI 12–30) for AFP ≥ 1000 ng/mL; 1-year and 5-year cumulative probabilities of OS were 88% (95%CI 84–92) vs. 67% (95%CI 43–91) and 43% (95%CI 37–49) vs. 1% (95%CI 0–13), respectively (Figure 2d).

Among other baseline variables, age at enrollment (HR 1.3; 95%CI 0.9–1.9; *p* = 0.038), tumor dimension ≥ 5 cm (HR 1.8; 95%CI 1.3–2.6; *p* = 0.001), Child–Pugh class B or C (vs. A) (HR 1.6; 95%CI 1.2–2.3; *p* = 0.004), BCLC stage A (vs. 0) (HR 2.1; 95%CI 1.5–3.0; *p* < 0.001) presence of PVT (HR 1.8; 95%CI 1.3–2.6; *p* = 0.001), and malignant PVT (HR 2.2; 95%CI 1.5–3.2; *p* < 0.001) were correlated with increased mortality at univariate analysis (Table 2). At the multivariate regression analysis, AFP ≥ 1000 ng/mL (HR 2.0; 95%CI 1.2–3.5; *p* = 0.009), tumor size ≥ 5 cm (HR 1.5; 95%CI 1.0–2.2; *p* = 0.034), Child–Pugh class B or C (vs. A) (HR 1.4; 95%CI 1.0–2.0; *p* = 0.042), BCLC stage A (vs. 0) (HR 1.8; 95%CI 1.2–2.6; *p* = 0.003), and malignant PVT (HR 2.3; 95%CI 1.2–4.4; *p* = 0.009) proved to be independent risk factors for mortality (Table 2).

The 1- and 5-year cumulative probabilities of DSS were 90% (95%CI, 86–94) and 50% (95%CI, 44–56), respectively, with a median of 58 months (95%CI, 47–69). A baseline AFP value ≥ 1000 ng/mL was found to be a risk factor also for DSS, both at the univariate and the multivariate regression analyses (HR 2.1; 95%CI 1.1–4.1; *p* = 0.019), along with tumor dimension ≥ 5 cm (HR 1.7; 95%CI 1.1–2.6; *p* = 0.012), Child–Pugh class B or C (HR 1.7; 95%CI 1.2–2.6; *p* = 0.007), BCLC stage A (vs. 0) (HR 1.9; 95%CI 1.2–2.9; *p* = 0.008), and malignant PVT (HR 2.2; 95%CI 1.1–4.5; *p* = 0.023).

### 3.3. Correlations between AFP and HCC Recurrence

**Figure 3:**
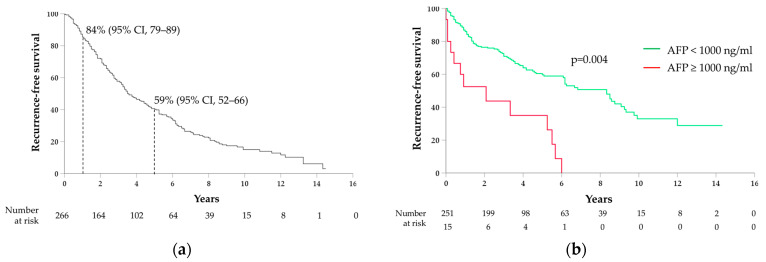
Kaplan–Meier curves for recurrence-free survival: (**a**) overall, with 1- and 5-year survival rates shown; (**b**) stratified by AFP cut-off of 1000 ng/mL.

During the follow-up, 116 patients (43.6%) experienced an HCC recurrence. The 1- and 5-year cumulative probabilities of RFS were 86% (95%CI, 82–90) and 80% (95%CI, 74–86), respectively, with a median RFS of 42 months (95%CI 26–58) (Figure 3a). At univariate analysis, tumor dimension ≥ 5 cm (HR 2.6; 95%CI 1.7–4.1; *p* < 0.001), BCLC stage A (vs. 0) (HR 2.2; 95%CI 1.2–4.0; *p* = 0.008), HCC grade 3 (vs. 1–2) (HR 1.4; 95%CI 0.8–2.4; *p* = 0.032), PVT (HR 2.4; 95%CI 1.5–3.7; *p* < 0.001), malignant PVT (HR 2.4; 95%CI 1.5–3.9; *p* < 0.001), and AFP ≥ 1000 ng/mL (HR 1.8; 95%CI 1.2–4.1; *p* = 0.021) were correlated with a reduced RFS. At multivariate regression analysis, tumor dimension ≥ 5 cm (HR 2.3; 95%CI 1.5–3.8; *p* = 0.001), BCLC stage A (vs. 0) (HR 2.4; 95%CI 1.2–4.8; *p* = 0.011), HCC grade 3 (vs. 1–2) (HR 1.7; 95%CI 1.0–2.8; *p* = 0.003), and AFP ≥ 1000 ng/mL (HR 2.0; 95%CI 1.0–3.7; *p* = 0.038) (Figure 3b) were confirmed to be independent risk factors for recurrence (Table 3).

Forty-one patients (15.4%) experienced a local HCC recurrence after treatment, with a median time to recurrence of 12 months (95%CI 2–22). AFP ≥ 1000 ng/mL (HR 2.8; 95%CI 1.4–7.3; *p* = 0.005) and HCC grade 3 (vs. 1–2) (HR 2.8; 95%CI 1.0–8.1; *p* = 0.047) were correlated with an increased risk of LR.

Development of a new intrahepatic HCC was observed in 77 (28.9%) patients, without correlations with the AFP cut-offs or other baseline clinical variables.

Twenty-eight patients developed at least one extrahepatic HCC metastasis during the follow-up. Patients with an AFP value ≥ 20 ng/mL showed an increased risk of developing HCC metastases over time compared with patients with AFP < 20 ng/mL (HR 3.5; 95%CI 1.6–7.8; *p* = 0.002). This correlation was also confirmed for the other higher AFP cut-offs. Other baseline factors associated with extrahepatic HCC recurrence were tumor dimension ≥ 5 cm (HR 4.3; 95%CI 1.9–10.1; *p* = 0.001), HCC grade 3 (vs. 1–2) (HR 3.5; 95%CI 1.4–9.1; *p* = 0.009), and malignant PVT (HR 3.6; 95%CI 1.5–8.6; *p* = 0.005).

### 3.4. Correlations between AFP and Baseline Variables

The AFP cut-offs were progressively correlated with other baseline variables at HCC diagnosis. A baseline AFP ≥ 20 ng/mL was associated with tumor dimension ≥ 5 cm (*p* = 0.008) and histological tumor grading G3 (*p* < 0.001). Starting from a value ≥ 400 ng/mL, AFP showed a correlation with Child–Pugh score B or C (*p* = 0.038). No further correlations were observed for AFP ≥ 1000 ng/mL (Table 4). The AFP absolute values were also correlated with cirrhosis etiology, without a difference in median AFP between patients with viral etiology vs. others (18 ng/mL vs. 13 ng/mL, respectively; *p* = 0.096).

**Figure 4:**
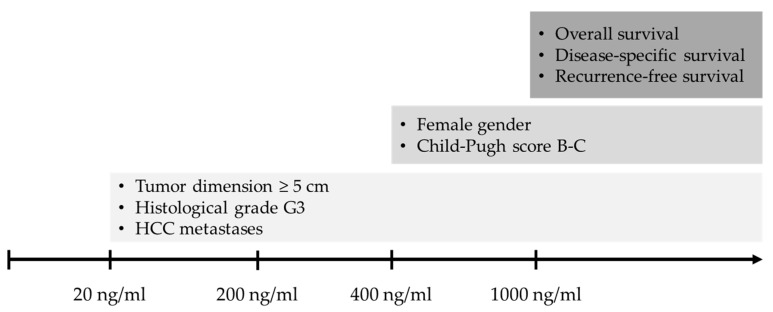
Summary figure showing the predictive power of the different AFP cut-offs towards outcomes and baseline clinical variables.

All correlations between AFP cut-offs and outcomes and baseline variables are summarized in Figure 4.

## 4. Discussion

In this study, we investigate the association between AFP, as measured at HCC diagnosis, and survival outcomes and HCC features in a cohort of patients with newly diagnosed HCC suitable for curative treatment. One-year and five-year OS rates in our cohort were 87% and 41%, respectively. This survival rate is higher than the 20% 5-year survival rate reported from the overall Italian nationwide cohort of HCC patients [32], while it is similar to the survival rates reported for the patients with HCC at very early and early stages (BCLC 0-A) [33,34]. This reflects the specific cohort we analyzed, which is composed only of patients suitable for curative treatment and is characterized by a relatively limited (<3 cm) median tumor dimension, which results from the strict adherence of our patients to surveillance programs and allowed most patients to be eligible for local ablation therapies.

Regarding our primary outcome, we found that a very high level of baseline AFP, specifically ≥1000 ng/mL, correlated with impaired survival, both considered overall and liver-related. In our cohort, the median OS in HCC patients with AFP in this range was 21 months, similar to what is reported in patients with unresectable HCC (BCLC B) [7,35]. AFP ≥ 1000 ng/mL also proved to be an independent risk factor for mortality in the multivariate Cox regression analysis. Our results agree with previous evidence from the literature. In a systematic review and meta-analysis, Hakeem et al. [27] analyzed a total of more than 12.000 patients and demonstrated better survival for patients with a preliver transplant AFP level < 1000 ng/mL. They also found that high pretransplant AFP was associated with poor tumor differentiation, in accordance with what we found in our cohort, where positive AFP was associated with histological HCC grade 3. More recently, Silva et al. [36] analyzed a large registry of more than 40.000 HCC patients and found that patients with elevated (200–1999 ng/mL) and highly elevated (≥2000 ng/mL) basal AFP levels had impaired OS, regardless of the HCC treatment plan. In a very recently published study, Yao et al. [37] assessed the predictive role of AFP in patients undergoing liver resection of early-stage HCC and showed that patients with high (400–999 ng/mL) and extremely high (≥1000 ng/mL) preoperative AFP were characterized by worse OS compared with patients with low (<400 ng/mL) AFP. Several other studies showed similar results [38,39,40,41,42].

We failed to find a discriminating role of the 400 ng/mL basal AFP cut-off toward survival outcomes. On the contrary, combined with previous literature evidence, our results seem to confirm the 1000 ng/mL cut-off as a reliable tool to identify patients with significantly different survival among those undergoing an HCC curative treatment, and its application in clinical practice in order to select patients needing a more aggressive follow-up and treatment approach may be considered.

In our cohort, AFP ≥ 1000 ng/mL was also associated with an increased risk of HCC recurrence and reduced recurrence-free survival. This result was expected and directly follows the correlation with survival, since HCC recurrence (intra- or extra-hepatic) is a major determinant of poor prognosis, and most patients in our cohort died of liver-related conditions. By differentiating between different patterns of recurrence, AFP ≥ 1000 ng/mL was correlated with local HCC recurrence after treatment, while AFP values ≥ 20 ng/mL with the development of metastases from HCC. These results also agree with previously published data [15,41,43,44,45]. In particular, numerous studies in the literature demonstrated that AFP is capable of activating the expression of metastasis-related factors through the PI3K/AKT179 pathway, therefore increasing the risk of metastasis development [28,46,47,48,49].

At the multivariate analysis, our results show that in addition to AFP ≥ 1000 ng/mL, tumor size ≥ 5 cm, Child–Pugh functional class B or C, BCLC stage A (early) compared with stage 0 (very early), and malignant PVT were independent risk factors for increased mortality. All these correlations agree with previous evidence. The 5 cm dimension cut-off has proved to predict more aggressive tumor behavior and prognosis in several studies [33,50,51,52], and the prognostic role of the Child–Pugh score in cirrhotic patients has been widely demonstrated [53,54,55]. Malignant PVT is an expression of advanced diseases and significantly affected survival in our cohort, similar to what has been shown in many previous studies [56,57,58,59,60].

In our study, the basal AFP value was significantly correlated with several clinical and HCC-related features at diagnosis. Regarding the size of the nodule, HCC with AFP ≥ 20 ng/mL showed larger dimensions, and statistical significance was maintained moving to groups with higher AFP cut-offs. The correlation between high values of AFP and HCC size ≥ 5 cm is widely reported [28,40,61,62]. Starting from the cut-off of 400 ng/mL, we observed that female patients and patients in more advanced functional classes (i.e., Child–Pugh B and C) were more represented. Also, these correlations are consistent with some studies in the literature [22,41,46,63,64,65], even though they are not confirmed by others [66,67,68]. Many previous studies reported a connection between PVT and neoplastic PVT, and higher values of AFP [46,69,70]. However, we did not find a correlation between these baseline variables and the various AFP cut-off groups, even at the highest 1000 ng/mL cut-off, a result that is in a certain sense unexpected, particularly considering that malignant PVT was a predictor of relevant outcomes, as described above; this could be due to the small number of patients with malignant PVT in our cohort, which yielded insufficient statistical power to find this connection. Finally, in relation to histological grading, our study showed a prevalence of HCC with poorly differentiated histological grade (G3) starting from AFP values ≥ 20 ng/mL, and this difference remained statistically significant progressing towards higher AFP cut-offs. These data are confirmed by numerous studies and could indicate that HCCs with elevated AFP values are more aggressive but also that a loss of cellular differentiation could lead to an increased production of AFP [71,72,73].

Our study has some limitations, of which the sample size and the retrospective nature of this study are the main ones. The sample size, although sufficient to demonstrate numerous correlations, was relatively small, especially for the highest AFP cut-offs, and some correlations could have not emerged. Moreover, the decision not to analyze a cohort of patients undergoing a single specific HCC treatment could be considered a limit of the study; however, this decision was driven by the purpose of assessing the predictive role of different AFP cut-offs in a wider, real-world-setting cohort, irrespective of the curative treatment performed. The retrospective nature of this study makes it necessary to validate our results on prospective cohorts. The strengths of this study were the homogeneity of the study cohort, with particular reference to regularity and adherence to the follow-up after HCC diagnosis and after treatment, the relevance of the outcomes analyzed, and the long-term follow-up that make these outcomes more robust and our results more reliable.

## 5. Conclusions

AFP is a simple and cost-saving biomarker of HCC. We demonstrated a predictive role of different AFP cut-offs, as assessed at HCC diagnosis, toward relevant outcomes in patients with HCC, mainly overall survival. We also found correlations between AFP and baseline features of the tumor. All these findings allow us to assess that a high basal AFP level correlates with more aggressive tumor behavior. Therefore, baseline AFP could help to immediately identify patients at higher risk of unfavorable disease evolution in order to refer them to a personalized therapeutic and follow-up approach, in accordance with the concept of precision medicine, with the aim of improving their quality of life and survival. Our results require validation in prospective and larger studies.

## Figures and Tables

**Table 1:** Demographic and clinical characteristics of the 266 patients analyzed.

	Total
Parameter	*n* = 266
Sex (F), n (%)	85 (32)
Age at HCC enrollment, years, median (range)	73 (45–87)
Etiology (viral vs. others), n (%)	
Viral	212 (80)
Others	54 (20)
Dimension, mm, median (range)	26.5 (12–150)
Diameter, n (%)	
≥5 cm	224 (84)
<5 cm	42 (16)
Child–Pugh score, n (%)	
A	215 (81)
B–C	51 (19)
BCLC stage	
0 (Very early)	60 (23)
A (Early)	206 (77)
HCC Grade ^1^ (1–2 vs. 3), n (%)	
1–2	201 (85)
3	36 (15)
PVT, n (%)	42 (16)
Malignant PVT, n (%)	37 (14)
Type of curative treatment, n (%)	
RFTA	201 (76)
PEI	48 (18)
Resection	29 (11)
TACE/TAE ^2^	39 (15)
Type of curative treatment, n (%)	
AFP cut-offs	91 (34)
≥20 ng/mL	31 (12)
≥200 ng/mL	21 (8)
≥400 ng/mL	15 (6)
≥1000 ng/ml	
HCC recurrence, n (%) ^3^	116 (44)
Local recurrence after treatment	41 (15)
New intrahepatic	77 (29)
Extrahepatic metastases	28 (11)
Dead at f-up end, n (%)	212 (80)
Disease-related death, n (%)	157 (59)
Follow-up time, months, median (range)	41.5 (1–174)

^1^ Available for 237 patients; ^2^ always in combination with RFTA or PEI; ^3^ some patients experienced more than one type of HCC recurrence; AFP: alpha-fetoprotein; BCLC: Barcellona Clinic Liver Cancer; HCC: hepatocellular carcinoma; PVT: portal vein thrombosis; RFTA: radiofrequency thermal ablation; PEI: percutaneous ethanol injection; TACE: transcatheter arterial chemoembolization; TAE: transarterial chemoembolization.

**Table 2:** Univariate and multivariate analysis of baseline predictors of overall survival.

Features	Univariate Analysis		Multivariate Analysis	
	HR (95%CI)	*p* Value ^1^	HR (95%CI)	*p* Value ^1^
Sex (F vs. M)	0.96 (0.72–1.28)	0.764		
Age at HCC enrollment	1.29 (0.87–1.92)	0.038	1.02 (1.00–1.04)	0.120
Etiology (viral vs. others)	1.19 (0.85–1.69)	0.314		
Diameter (≥5 cm vs. <5 cm)	1.79 (1.26–2.55)	0.001	1.50 (1.03–2.18)	0.034
Child-Pugh score (B–C vs. A)	1.63 (1.17–2.27)	0.004	1.41 (0.99–2.01)	0.042
BCLC stage (A vs. 0)	2.07 (1.46–2.95)	<0.001	1.79 (1.22–2.62)	0.003
HCC Grade (3 vs. 1–2)	1.43 (0.98–2.09)	0.061		
PVT (yes vs. no)	1.84 (1.29–2.63)	0.001	1.03 (0.56–1.89)	0.918
Malignant PVT (yes vs. no)	2.20 (1.51–3.21)	<0.001	2.34 (1.24–4.44)	0.009
Type of treatment (locoregional vs. surgical)	1.29 (0.84–2.00)	0.247		
AFP ≥ 20 ng/mL (vs. < 20 ng/mL)	1.17 (0.88–1.55)	0.285		
AFP ≥ 200 ng/mL (vs. < 200 ng/mL)	1.27 (0.82–1.99)	0.287		
AFP ≥ 400 ng/mL (vs. < 400 ng/mL)	1.11 (0.67–1.83)	0.688		
AFP ≥ 1000 ng/mL (vs. < 1000 ng/mL)	2.29 (1.35–3.89)	0.002	2.20 (1.28–3.78)	0.004

^1^ Cox regression analysis; CI: confidence interval; AFP: alpha-fetoprotein; BCLC: Barcellona Clinic Liver Cancer; HCC: hepatocellular carcinoma; HR: hazard ratio; PVT: portal vein thrombosis.

**Table 3:** Univariate and multivariate analysis of baseline predictors of recurrence-free survival.

Features	Univariate Analysis		Multivariate Analysis	
	HR (95%CI)	*p* Value ^1^	HR (95%CI)	*p* Value ^1^
Sex (F vs. M)	0.69 (0.46–1.05)	0.091		
Age at HCC enrollment	1.01 (0.99–1.04)	0.207		
Etiology (viral vs. others)	0.88 (0.57–1.35)	0.560		
Diameter (≥5 cm vs. <5 cm)	2.60 (1.67–4.07)	<0.001	2.36 (1.45–3.83)	0.001
Child-Pugh score (B–C vs. A)	1.47 (0.94–2.29)	0.092		
BCLC stage (A vs. 0)	2.21 (1.24–3.96)	0.008	2.41 (1.22–4.75)	0.011
HCC Grade (3 vs. 1–2)	1.41 (0.84–2.38)	0.032	1.65 (1.04–4.67)	0.003
PVT (yes vs. no)	2.39 (1.54–3.71)	<0.001	1.55 (0.60–4.01)	0.363
Malignant PVT (yes vs. no)	2.40 (1.48–3.88)	<0.001	1.69 (0.62–4.61)	0.304
Type of treatment (locoregional vs. surgical)	1.41 (0.81–2.48)	0.229		
AFP ≥ 20 ng/mL (vs. < 20 ng/mL)	1.23 (0.84–1.79)	0.283		
AFP ≥ 200 ng/mL (vs. < 200 ng/mL)	1.04 (0.54–1.99)	0.412		
AFP ≥ 400 ng/mL (vs. < 400 ng/mL)	1.97 (1.03–2.17)	0.104		
AFP ≥ 1000 ng/mL (vs. < 1000 ng/mL)	1.82 (1.20–4.13)	0.021	1.95 (1.04–3.68)	0.038

^1^ Cox regression analysis; CI: confidence interval; AFP: alpha-fetoprotein; BCLC: Barcellona Clinic Liver Cancer; HCC: hepatocellular carcinoma; HR: hazard ratio; PVT: portal vein thrombosis.

**Table 4:** Correlation between the increasing AFP cut-offs and the baseline clinical variables.

	AFP Cut-Offs											
	20 ng/mL			200 ng/mL			400 ng/mL			1000 ng/mL		
Parameter	<	≥	*p* ^2^	<	≥	*p* ^2^	<	≥	*p* ^2^	<	≥	*p* ^2^
Sex (F), %	30	32	0.368	31	39	0.102	30	50	0.045	31	47	0.020
Age at HCC enrollment, years, median	73	72	0.672	73	72	0.753	73	72	0.903	73	73	0.669
Viral etiology, %	76	87	0.073	79	89	0.242	79	91	0.201	79	87	0.743
Diameter ≥ 5 cm, %	11	24	0.006	14	30	0.027	15	29	0.032	15	33	0.045
Child-Pugh score A, %	82	79	0.566	83	63	0.061	82	62	0.022	83	53	0.012
BCLC A, %	75	81	0.354	76	89	0.153	77	86	0.426	77	87	0.532
HCC Grade ^1^ 1–2, %	84	69	<0.001	88	52	<0.001	87	53	<0.001	88	36	<0.001
PVT, %	13	20	0.178	16	19	0.612	15	19	0.670	16	20	0.713
Malignant PVT, %	12	18	0.192	14	11	0.713	14	9	0.748	14	13	0.947

^1^ Available for 237 patients; ^2^ Mann–Whitney analysis or Fisher’s exact test; AFP: alpha-fetoprotein; BCLC: Barcellona Clinic Liver Cancer; HCC: hepatocellular carcinoma; PVT: portal vein thrombosis.

## Data Availability

The research data are stored in an institutional repository and will be shared upon request to the corresponding author.
